# Is Lymphadenectomy Reasonable for Elderly Intrahepatic Cholangiocarcinoma Patients?

**DOI:** 10.1007/s11605-023-05846-y

**Published:** 2023-10-02

**Authors:** Qianyi Lin, Jianjun Chen, Kangde Li, Junxing Yang, Xiaofeng Luo, Qi Cai, Weihong Lin, Guanjing Peng, Dexiong Chen, Chunhong Qin, Tao He, Zhenlong Wang

**Affiliations:** 1 Sixth Department of General Surgery, Central People’s Hospital of Zhanjiang, Zhanjiang Central Hospital, Guangdong Medical University, Zhanjiang, Guangdong Province China; 2 Second Department of General Surgery, Central People’s Hospital of Zhanjiang, Zhanjiang Central Hospital, Guangdong Medical University, Zhanjiang, Guangdong Province China; 3 Seventh Department of General Surgery, Central People’s Hospital of Zhanjiang, Zhanjiang Central Hospital, Guangdong Medical University, Zhanjiang, Guangdong Province China

**Keywords:** Intrahepatic cholangiocarcinoma, Elderly, Lymphadenectomy, Perioperative mortality, Overall survival

## Abstract

**Background:**

In this study, we aimed to determine the impact of lymphadenectomy (LND) on clinical outcomes in ICC patients aged ≥ 70 years.

**Methods:**

Four hundred and three eligible patients diagnosed with ICC who underwent hepatectomy between 2004 and 2019 were enrolled in the Surveillance, Epidemiology, and End Results database. The impact of LND on perioperative mortality and overall survival (OS) as well as the optimal total number of lymph nodes examined (TNLE) was estimated.

**Results:**

One hundred thirty-nine pairs of patients were matched by propensity score matching. Perioperative mortality was comparable between the LND and non-LND (nLND) groups (0.7% vs. 2.9%, *P* = 0.367). The median OS in the LND group was significantly longer (44 vs. 32 months, *P* = 0.045) and LND was identified as an independent protective factor for OS by multivariate analysis (HR 0.65, 95% CI 0.46–0.92, *P* = 0.014). Patients with the following characteristics were potential beneficiaries of LND: white, female, no/moderate fibrosis, tumor size > 5 cm, solitary tumor, and localized invasion (all *P* < 0.05). TNLE ≥ 6 had the greatest discriminatory power for identifying lymph node metastasis (area under the curve, 0.704, Youden index, 0.365, *P* = 0.002). Patients with pathologically confirmed lymph node metastasis are likely to benefit from adjuvant therapy (40 months vs. 4 months, *P* = 0.052).

**Conclusions:**

Advanced age (≥ 70 years) was not a contraindication for LND, which facilitates accurate nodal staging and guides postoperative management. Appropriately selected elderly populations could benefit from LND.

**Supplementary Information:**

The online version contains supplementary material available at 10.1007/s11605-023-05846-y.

## Background

Primary liver cancer ranks as the sixth most commonly diagnosed cancer and the third leading cause of cancer-related death worldwide.^1^ Intrahepatic cholangiocarcinoma (ICC) is the second most common primary liver cancer with a globally increasing age of incidence.^2^ The average age at first diagnosis for ICC patients is 70 in the USA.^3^ At present, surgical resection remains the mainstay of curative procedures for ICC patients, including elderly patients.^4^ Due to the high prevalence and extremely negative prognostic effect of lymph node metastasis (LNM) in ICC,^5^ routine lymphadenectomy (LND) is recommended by the guidelines of both the European Association for the Study of the Liver (EASL)^6^ and the National Comprehensive Cancer Network (NCCN).^7^ However, the practice rate of LND varies among different centers,^8, 9^ ranging from 27 to 100%.^10, 11^ According to an international multicenter study (*n* = 1084), from 2000 to 2015, the average practice rate of LND was merely 49.4%.^12^ In addition, the minimal number of lymph nodes (LNs) harvested is controversial, with a real-world practical median of 2–4.^13–15^ The low practice rate of LND and conservative LND strategies may be attributed to the increased incidence of complications and the controversial effect on long-term survival.^16, 17^

It was previously indicated that not all ICC patients could benefit from LND.^16, 18, 19^ Furthermore, the potential benefits of LND on long-term survival should be weighed against the risk of postoperative complications related to the procedure itself; elderly ICC patients who underwent complex surgical procedures were more likely to suffer severe complications due to poor performance status,^20, 21^ and LND may lead to an increase in operative time^22^ and blood loss^23^ and result in subsequent postoperative morbidity such as bile leakage, hemorrhage, and even perioperative death.^24, 25^

Whether LND is reasonable for elderly (≥ 70 years) ICC patients and the optimal total number of LNs examined (TNLE) remains unclear. Data regarding the impact of LND on clinical outcomes for elderly ICC patients are sporadic. To our knowledge, propensity score matching (PSM) analysis,^26^ which could control the selection bias of retrospective studies, has not yet been conducted in such studies. The current study, as such, was designed to compare the perioperative mortality and overall survival of ICC patients aged ≥ 70 years who did or did not undergo LND by PSM and to determine the optimal threshold of TNLE for discriminating LNM using a national database of ICC patients.

## Patients and Methods

### Patients and Data Collection

Patients who underwent cancer-directed surgery (codes 20–75, excluding liver transplantation) for ICC between 2000 and 2019 were identified in the SEER database using the 3rd edition International Classification of Disease for Oncology-3 codes by software SEER.Stat 8.4.0.1. Patients with ICC were identified using the primary site code for liver (22.0) and intrahepatic bile duct (22.1) and histology code for malignant neoplasm (8000), malignant tumor cells (8001), carcinoma (8010), undifferentiated carcinoma (8020), adenocarcinoma (8140), and cholangiocarcinoma (8160).^14^ Patients with recurrent ICC, concomitant or history of other malignancies, distant metastases, performance of preoperative or intraoperative anticancer therapies, age less than 70 years, missing information on LND, or who were lost to follow-up within 30 days after surgery were excluded. In turn, a total of 403 eligible patients were finally included in the analytic cohort. Clinically relevant information including age, sex, race, fibrosis, tumor size, tumor number, histologic grade, vascular invasion, invasive extent, nodal status (categorized as positive, negative, or unstaged), TNLE, and adjuvant therapy was extracted. The extent of LND was not clearly documented in the SEER database, and LND is generally defined as the presence of lymph node examination, as previously reported.^8, 12, 27, 28^ To further clarify the clinical significance of TNLE, patients with LND were further classified into adequate-LND (AD-LND) and inadequate-LND (inAD-LND) according to the optimal threshold of TNLE for identifying LNM. The study was performed in accordance with principles of the Declaration of Helsinki and approved by the Institutional Review Board of Zhanjiang Central Hospital, Guangdong Medical University.

### Statistical Analysis

Continuous variables are summarized as medians with interquartile ranges (IQRs) and were compared by the Mann‐Whitney *U* test. Qualitative variables were grouped and analyzed by the Pearson chi-squared test or Fisher’s exact test, as appropriate. The propensity scores for all patients were estimated by a logistic regression model using preoperative and intraoperative characteristics as covariates: age, sex, race, tumor size, tumor number, and invasive extent. A one-to-one nearest-neighbor matching algorithm with a caliper of 0.1 and without replacement was used. Corresponding standardized mean differences (SMDs) of matched characteristics were estimated. An SMD of < 0.1 indicates very small differences, values between 0.1 and 0.3 indicate small differences, values between 0.3 and 0.5 indicate moderate differences, and values > 0.5 indicate considerable differences.^29^ Receiver-operating characteristic curve (ROC) analysis was conducted to investigate the optimal threshold of TNLE for detecting LNM. Logistic regression was used to identify factors associated with LNM. Kaplan‒Meier curves were used to estimate the median survival of patients who survived the perioperative period (30 days after surgery), and the log-rank test was used to assess differences in overall survival (OS). Potential variables associated with OS were univariately analyzed utilizing Cox proportional hazards regression. Variables with a *P* value of less than 0.10 in univariate analysis were included in multivariate analysis. Two-tailed *P* values less than 0.05 were considered statistically significant. Statistical computation was performed by R software (version 4.2.3, R Foundation for Statistical Computing, Vienna, Austria) with packages “MatchIt,” “pROC,” and “survival.”

## Results

### Baseline Characteristics

**Fig. 1:**
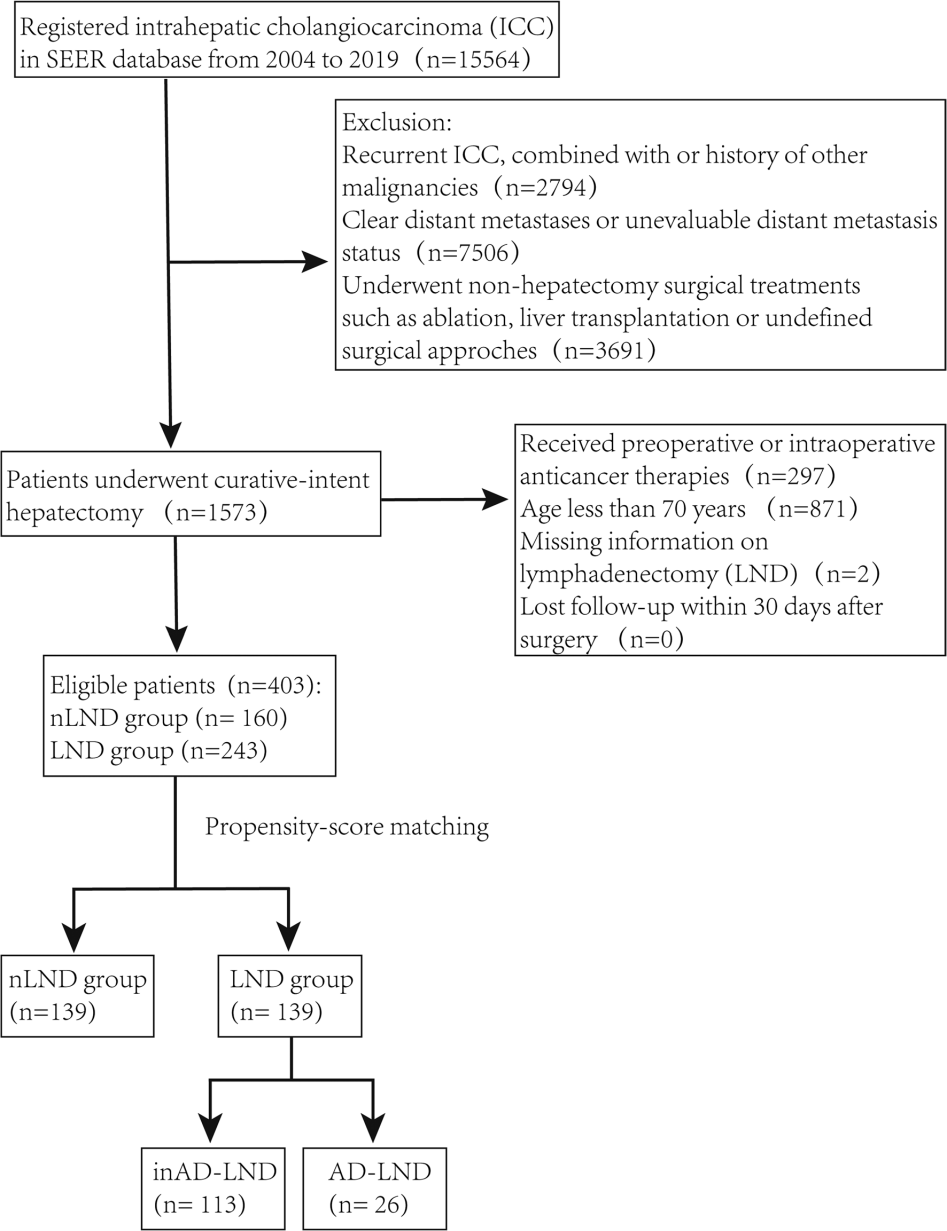
Flow chart of patient enrollment. One patient died perioperatively in the adequate-lymphadenectomy (AD-LND) group, which was excluded from the survival analysis

The patient enrollment flow diagram is shown in Fig. 1. A total of 403 patients with a median age of 75 years were enrolled in this study, including 183 (45.4%) males and 220 (54.6%) females. Baseline characteristics are listed in Table 1. Two hundred forty-three patients underwent LND with a median TNLE of three, while 160 patients did not. All preoperative and intraoperative variables were balanced except for the extent of tumor invasion (Table 1). After PSM, 139 pairs of patients were matched, and clinicopathological variables between the two groups were relatively balanced.

**Table 1:** Clinicopathological characteristics before and after PSM

	Before PSM				After PSM			
Variable	nLND	LND	*P* value	SMD	nLND	LND	*P* value	SMD
	(*n* = 160)	(*n* = 243)			(*n* = 139)	(*n* = 139)		
Age (years, median [IQR])	75.0 [72.0, 78.3]	75.0 [72.0, 77.0]	0.320	0.115	75.0 [72.0, 79.0]	75.0 [72.0, 77.0]	0.398	0.134
Sex (%)								
Male	76 (47.5)	107 (44.0)	0.561	0.070	64 (46.0)	68 (48.9)	0.719	0.058
Female	84 (52.5)	136 (56.0)			75 (54.0)	71 (51.1)		
Race (%)								
White	132 (82.4)	200 (82.3)	0.997	0.007	113 (81.3)	116 (83.5)	0.868	0.064
Black	6 (3.8)	9 (3.7)			6 (4.3)	6 (4.3)		
Other	22 (13.8)	34 (14.0)			20 (14.4)	17 (12.2)		
Fibrosis (%)								
No/moderate	56 (35.0)	76 (31.3)	0.666	0.092	45 (32.4)	46 (33.1)	0.596	0.122
Advanced/severe	7 (4.4)	9 (3.7)			6 (4.3)	3 (2.2)		
Unclear	97 (60.6)	158 (65.0)			88 (63.3)	90 (64.7)		
Tumor size (%)								
≤ 5 cm	74 (46.2)	110 (45.3)	0.923	0.041	66 (47.5)	65 (46.8)	0.928	0.047
> 5 cm	80 (50.0)	122 (50.2)			70 (50.4)	70 (50.4)		
Unclear	6 (3.8)	11 (4.5)			3 (2.2)	4 (2.9)		
Tumor number (%)								
Solitary	138 (86.2)	223 (91.8)	0.108	0.177	124 (89.2)	122 (87.8)	0.851	0.045
Multiple	22 (13.8)	20 (8.2)			15 (10.8)	17 (12.2)		
Histologic grade (%)								
Well/moderately	86 (53.8)	157 (64.6)	0.040	0.256	84 (60.4)	85 (61.2)	0.991	0.016
Poorly/undifferentiated	52 (32.5)	68 (28.0)			43 (30.9)	42 (30.2)		
Unclear	22 (13.7)	18 (7.4)			12 (8.6)	12 (8.6)		
Invasive extent (%)								
Localized	123 (76.9)	134 (55.1)	< 0.001	0.471	102 (73.4)	103 (74.1)	0.999	0.016
Regional	37 (23.1)	109 (44.9)			37 (26.6)	36 (25.9)		
Vascular invasion (%)								
No	81 (50.6)	91 (37.4)	0.004	0.350	66 (47.5)	67 (48.2)	0.987	0.019
Yes	33 (20.7)	42 (17.3)			28 (20.1)	27 (19.4)		
Unclear	46 (28.7)	110 (45.3)			45 (32.4)	45 (32.4)		
Nodal status (%)								
Negative	-	188 (77.4)	< 0.001	0.765	-	119 (85.6)	< 0.001	0.580
Positive	-	55 (22.6)			-	20 (14.4)		
Unstaged	160 (100.0)	-			139 (100.0)	0 (0.0)		
TNLE (median [IQR])	-	3.0 [2.0, 6.0]	< 0.001	0.792	-	3.0 [2.0, 6.0]	< 0.001	1.424
Adjuvant chemotherapy (%)								
No/unknown	119 (74.4)	157 (64.6)	0.051	0.213	101 (72.7)	100 (71.9)	0.999	0.016
Yes	41 (25.6)	86 (35.4)			38 (27.3)	39 (28.1)		
Adjuvant radiotherapy (%)								
No/unknown	143 (89.4)	210 (86.4)	0.468	0.091	124 (89.2)	124 (89.2)	0.999	< 0.001
Yes	17 (10.6)	33 (13.6)			15 (10.8)	15 (10.8)		

Values are presented as medians (IQRs) for quantitative variables and n (%) for categorical variables

Abbreviations: *PSM*, propensity score matching; *nLND*, non-lymphadenectomy; *LND*, lymphadenectomy; SMD, standardized mean differences; *IQR*, interquartile range; *TNLE*, total number of lymph nodes examined

### Correlation of TNLE and LNM

**Fig. 2:**
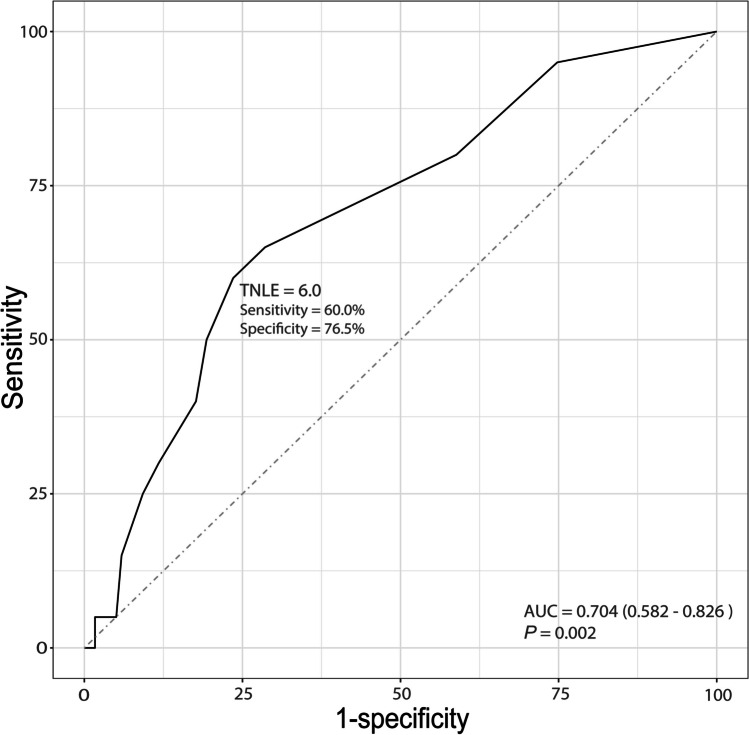
Receiver operative characteristics (ROC) analysis of identifying the optimal total number of lymph nodes examined (TNLE) for detecting LNM

ROC analysis indicated that TNLE ≥ 6 had the greatest discriminatory power relative to the diagnosis of LNM (area under the curve [AUC], 0.704, Youden index, 0.365) (Fig. 2). In addition, on multivariable logistic analysis, TNLE ≥ 6 (Ref. TNLE < 6, OR 6.49, 95% CI 1.98–21.28, *P* = 0.002) and vascular invasion (OR 4.62, 95% CI 1.06–20.12, *P* = 0.042) were significantly associated with pathologic LNM (Table 2).

**Table 2:** Univariate and multivariate logistic regression analysis of relative factors of lymph node metastases

Variable	Univariate		Multivariate	
	OR (95% *CI*)	*P* value	OR (95% *CI*)	*P* value
Age				
≤ 80	1.87 (0.55–6.41)	0.316		
> 80				
Sex				
Male	Ref			
Female	0.46 (0.17–1.24)	0.126		
Race				
White	Ref		Ref	
Black	1.46 (0.16–13.40)	0.739	4.89 (0.37–63.80)	0.226
Other	3.04 (0.93–9.91)	0.066	3.29 (0.74–14.74)	0.119
Fibrosis				
No/moderate	Ref		Ref	
Advanced/severe	7.17 (0.50–103.55)	0.148	7.75 (0.29–205.36)	0.221
Unclear	3.10 (0.85–11.25)	0.086	4.47 (1.03–19.49)	0.046
Tumor size				
≤ 5 cm	Ref			
> 5 cm	1.04 (0.39–2.74)	0.942		
Unclear	2.07 (0.19–22.19)	0.546		
Tumor number				
Solitary	Ref			
Multiple	0.34 (0.04–2.71)	0.307		
Histologic grade				
Well/moderately	Ref		Ref	
Poorly/undifferentiated	2.64 (0.98–7.11)	0.055	2.95 (0.90–9.69)	0.074
Unclear	0.77 (0.09–6.65)	0.810	0.47 (0.05–4.82)	0.524
Vascular invasion				
No	Ref		Ref	
Yes	3.54 (0.98–12.82)	0.054	4.62 (1.06–20.12)	0.042
Unclear	3.10 (0.96–9.97)	0.058	3.13 (0.77–12.76)	0.112
TNLE				
< 6	Ref		Ref	
≥ 6	4.87 (1.81–13.12)	0.002	6.49 (1.98–21.28)	0.002

Abbreviations: *TNLE*, total number of lymph nodes examined

### Perioperative Mortality

With surgery, a total of 11 patients died, for an overall perioperative mortality rate of 2.7% in the entire cohort. The incidence of perioperative mortality was comparable among the LND (*n* = 6, 2.5%) and nLND (*n* = 5, 3.1%) (*P* = 0.934) groups. After PSM, the total perioperative mortality was 1.8% (*n* = 5). No obvious significant difference was observed between the LND and nLND groups (0.7% vs. 2.9%, *P* = 0.367).

### Long-term Survival

**Fig. 3:**
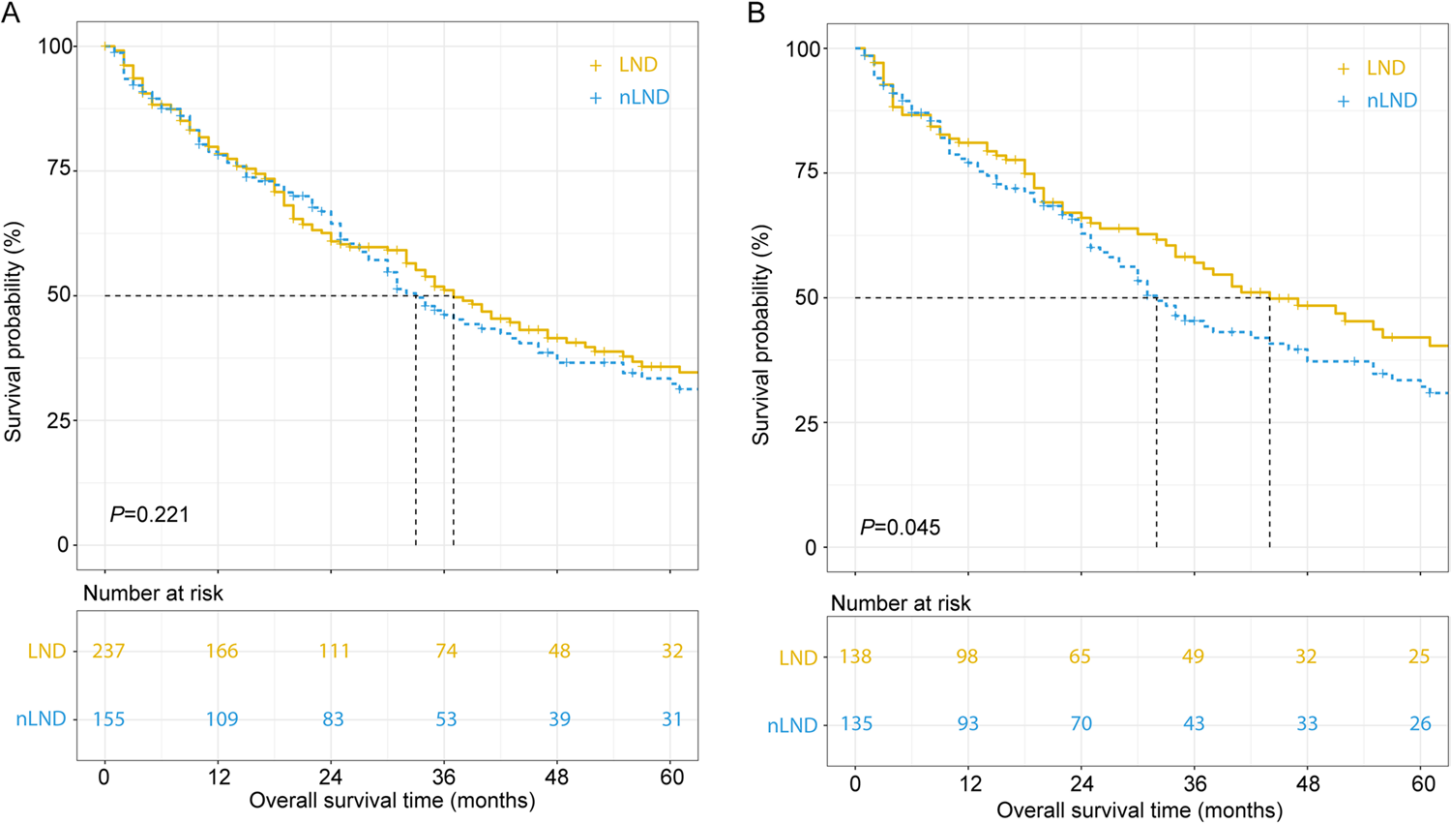
Overall survival of patients receiving lymphadenectomy (LND) or not before (**A**) and after propensity score matching (**B**)

Before PSM, 224 patients (57.1%) died within the follow-up period, with median OS and 1-year, 3-year, and 5-year survival rates of 35 months, 78.3%, 49.0%, and 34.3%, respectively. The median OS was comparable between the LND and nLND groups (median OS, 37 vs. 33 months, *P* = 0.221, Fig. 3A). In the multivariable analysis, male sex (Ref. female, HR 1.42, 95% CI 1.08–1.86, *P* = 0.010), positive nodal status (Ref. negative, HR 2.52, 95% CI 1.61–3.94, *P* < 0.001), and unstaged nodal status (without LND) (Ref. negative, HR 1.51, 95% CI 1.12–2.03, *P* = 0.006) were independently associated with worse OS among patients who underwent curative-intent resection for ICC.

After PSM, 155 (56.7%) patients died within the follow-up period. The median OS was 37 months, with 1-year, 3-year, and 5-year survival rates of 79.1%, 50.9%, and 36.8%, respectively. The median OS in the LND group was significantly longer than that in the nLND group (44 vs. 32 months, *P* = 0.045; Fig. 3B). After adjusting for sex, tumor number, invasive extent, and nodal status, LND was significantly identified as a protective factor for OS (HR 0.65, 95% CI 0.46–0.92, *P* = 0.014, Table 3). Interestingly, compared with inAD-LND, no significant survival improvement brought by AD-LND was observed either in the PSM cohort (mOS, 41 vs. 47 months, *P* = 0.612) or in the populations with negative nodal status (mOS, 40 vs. 55 months, *P* = 0.402), whereas AD-LND could prolong the OS of patients with LNM (mOS, 56 vs. 9 months, *P* = 0.043, supplementary 1).

**Table 3:** Univariate and multivariate analysis of OS before and after PSM

	Before PSM				After PSM			
	Univariate analysis		Multivariate analysis		Univariate analysis		Multivariate analysis	
	HR (95% *CI*)	*P* value	HR (95% *CI*)	*P* value	HR (95% *CI*)	*P* value	HR (95% *CI*)	*P* value
Age					1.26 (0.79–2.00)	0.338		
≤ 80 years	Ref							
> 80 years	1.03 (1.00–1.06)	0.295						
Sex								
Male	Ref		Ref		Ref		Ref	
Female	0.67 (0.52–0.89)	0.004	0.70 (0.54–0.92)	0.010	0.66 (0.48–0.91)	0.012	0.70 (0.51–0.97)	0.034
Race								
White	Ref				Ref			
Black	0.87 (0.41–1.85)	0.718			0.53 (0.20–1.44)	0.215		
Other	1.05 (0.71–1.56)	0.798			1.26 (0.80–1.98)	0.316		
Fibrosis								
No/moderate	Ref				Ref			
Advanced/severe	1.24 (0.66–2.34)	0.495			1.02 (0.44–2.38)	0.958		
Unclear	0.85 (0.65–1.12)	0.267			0.94 (0.67–1.33)	0.742		
Tumor size								
≤ 5 cm	Ref				Ref			
> 5 cm	1.19 (0.91–1.56)	0.201			1.17 (0.84–1.61)	0.354		
Unclear	1.01 (0.51–2.01)	0.978			1.77 (0.71–4.39)	0.221		
Tumor number								
Solitary	Ref		Ref		Ref		Ref	
Multiple	0.67 (0.45–1.00)	0.050	0.68 (0.45–1.02)	0.062	0.64 (0.40–1.01)	0.056	0.65 (0.41–1.04)	0.072
Histologic grade								
Well/moderately	Ref		Ref		Ref			
Poorly/undifferentiated	1.22 (0.91–1.63)	0.194	1.20 (0.89–1.61)	0.230	1.31 (0.92–1.86)	0.134		
Unclear	1.54 (1.03–2.31)	0.036	1.55 (1.03–2.34)	0.035	1.48 (0.88–2.50)	0.137		
Invasive extent								
Localized	Ref		Ref		Ref		Ref	
Regional	1.57 (1.20–2.06)	0.001	1.22 (0.88–1.69)	0.243	1.68 (1.20–2.37)	0.003	1.37 (0.93–2.03)	0.113
Vascular invasion								
No	Ref				Ref			
Yes	1.26 (0.89–1.78)	0.199			1.10 (0.74–1.64)	0.648		
Unclear	1.12 (0.83–1.51)	0.470			1.04 (0.72–1.50)	0.843		
Nodal status								
Negative	Ref		Ref		Ref		Ref	
Positive	2.74 (1.87–4.03)	< 0.001	2.52 (1.61–3.94)	< 0.001	2.71 (1.49–4.92)	0.001	2.03 (1.03–3.99)	0.040
Unstaged	1.48 (1.11–1.99)	0.008	1.51 (1.12–2.03)	0.006	1.60 (1.14–2.26)	0.007	–	–
Adjuvant chemotherapy								
No/unknown	Ref				Ref			
Yes	0.92 (0.69–1.24)	0.583			0.93 (0.65–1.35)	0.716		
Adjuvant radiation								
No/unknown	Ref				Ref			
Yes	1.26 (0.87–1.84)	0.223			1.17 (0.72–1.89)	0.532		
Lymphadenectomy								
No	Ref				Ref		Ref	
Yes	0.85 (0.65–1.10)	0.221			0.72 (0.52–0.99)	0.045	0.65 (0.46–0.92)	0.014

Abbreviations: *OS*, overall survival; *PSM*, propensity score matching

### Subgroup Analysis of Overall Survival Stratified by Risk Factors

**Fig. 4:**
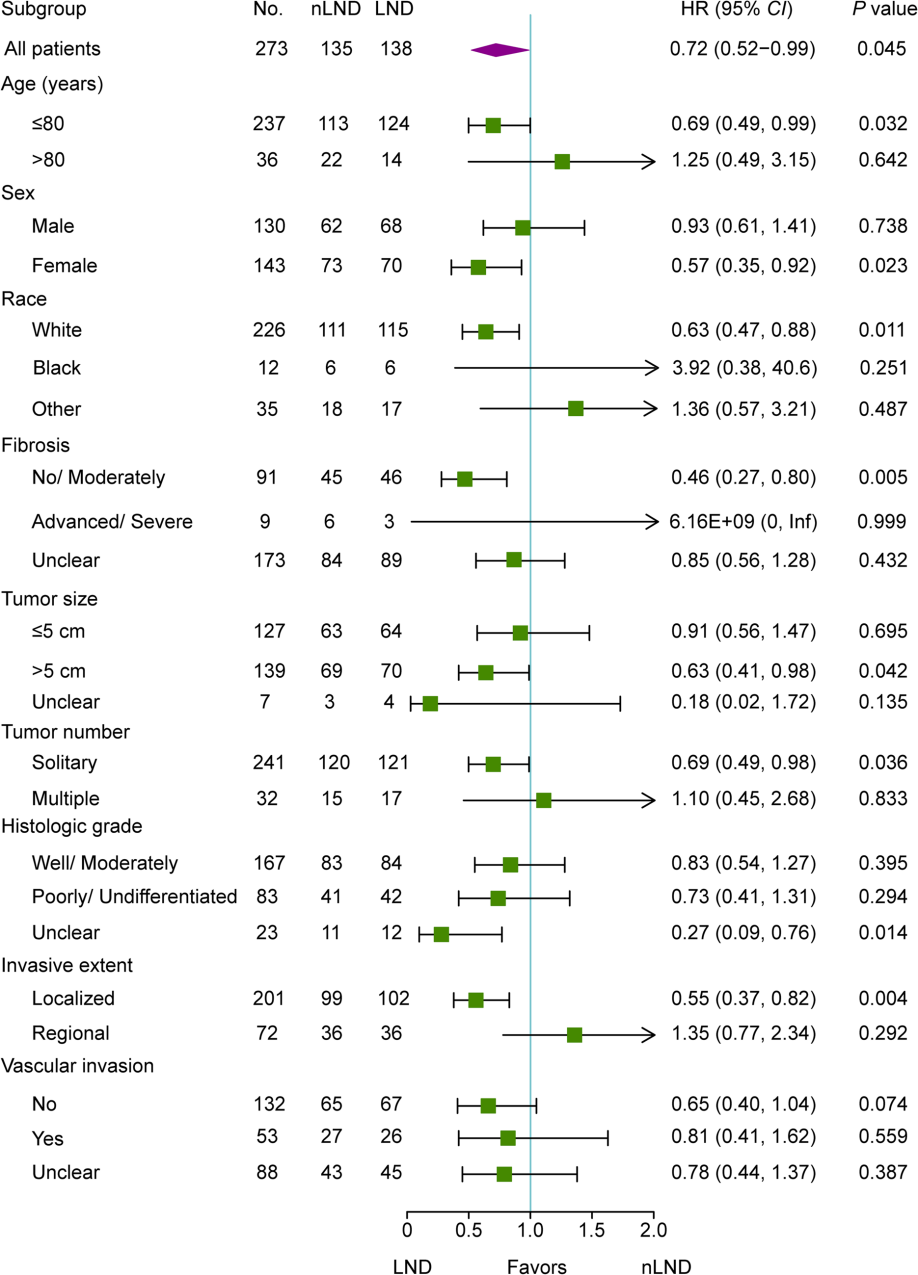
Forest plot of subgroup analysis stratified by risk factors in the matched cohort. nLND, non-lymphadenectomy; LND, lymphadenectomy

Subgroup analysis showed that patients with poor status or advanced tumor characteristics, including extremely advanced age (> 80 years), advanced/severe fibrosis, multiple tumors, regional invasion, and vascular invasion, could not benefit from LND (all *P* > 0.05, Fig. 4). Potential beneficiaries comprised the following characteristics: white, female, no/ moderate fibrosis, tumor size > 5 cm, solitary tumor, and localized invasion (all *P* < 0.05, Fig. 4). The survival benefit of LND in patients without evidence of vascular invasion was marginally significant (*P* = 0.074, Fig. 4).

### Effects of LND on Postoperative Management

**Fig. 5:**
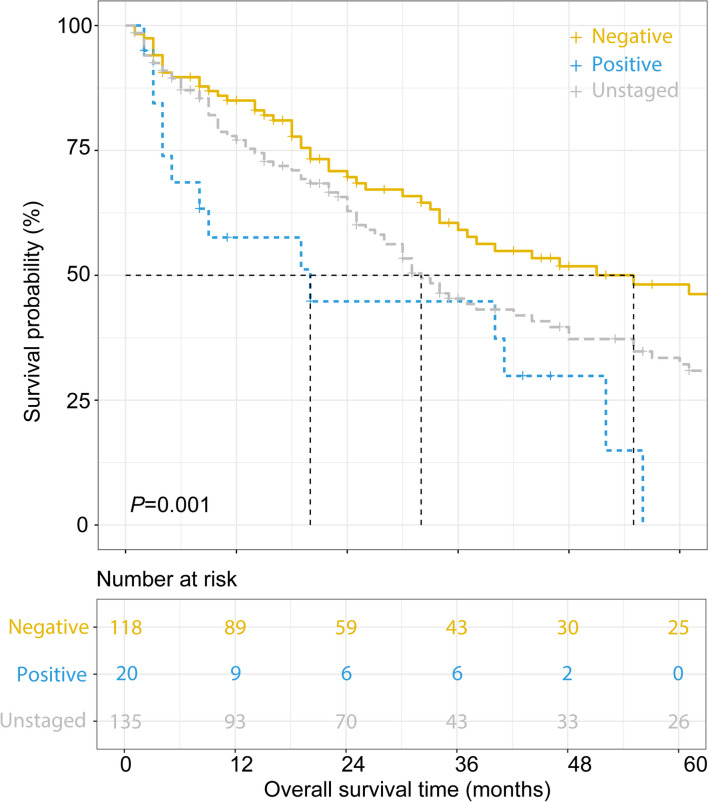
Overall survival of patients with negative, positive, or unstaged nodal status

**Fig. 6:**
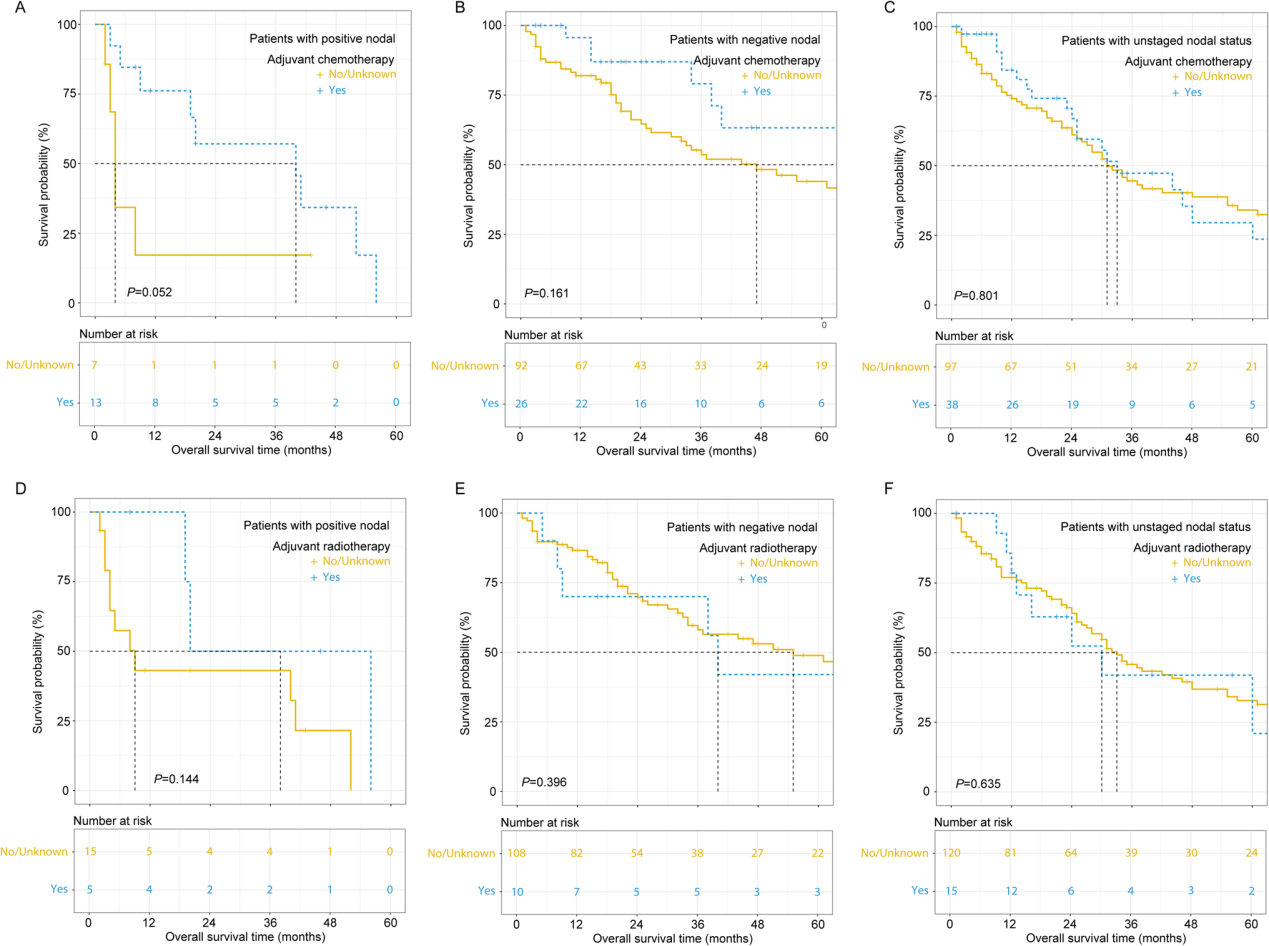
Overall survival of patients who received postoperative chemotherapy (**A**–**C**) or radiotherapy according to nodal status (**D**–**F**)

In the matched cohort, patients with unstaged nodal status showed superior OS to those with LNM but inferior OS to those with negative LNs (32 vs. 20 vs. 55 months, *P* = 0.001, Fig. 5). Receiving adjuvant chemotherapy could potentially improve the OS of patients with LNM (mOS, 40 vs. 4 months, *P* = 0.052, Fig. 6A). Nevertheless, postoperative chemotherapy could not prolong the OS of patients with negative nodal status (94.0 vs. 47.0 months, *P* = 0.161, Fig. 6B) or unstaged nodal status (33.0 vs. 31.0 months, *P* = 0.801, Fig. 6C). No significant survival benefit was observed with adjuvant radiotherapy regardless of LN status (all *P* > 0.05, Fig. 6D–F).

## Discussion

Whether LND is beneficial for elderly (≥ 70 years) ICC patients who undergo curative-intent hepatectomy, as well as minimal TNLE, remains unclear. The current study controlled baseline characteristics bias by PSM to evaluate the clinical effect of LND on elderly surgery candidates with ICC and demonstrated that an appropriately selected population could benefit from LND without increasing perioperative mortality. TNLE ≥ 6 had the greatest discriminatory power to identify LNM and guide postoperative management.

Elderly patients account for nearly half of the surgical practice for ICC.^28^ The poor general condition of elderly patients naturally causes a preconceived assumption that elderly patients cannot tolerate complex or difficult procedures.^30^ As the mainstay procedure to treat primary liver malignancies, the safety of hepatectomy in elderly patients has aroused controversy. In a large cohort with 27,094 candidates, the rate of mortality in patients who underwent hepatectomy increased notably with age but subsequently reached a plateau in septuagenarians.^4, 31^ By considering aging risk and selecting appropriate procedures, good outcomes after complex surgery could be attained even in septuagenarians.^31^ Due to the high prevalence and extremely negative prognostic effect of LNM, LND is currently widely recommended as a standardized procedure in curative surgery for ICC.^32^ Nevertheless, a meta-analysis indicated that LND markedly increased the risk of morbidity with an elevated incidence of postoperative complications and brought no obvious survival benefit.^16^ Therefore, the clinical value of LND is basically considered a procedure to accurately stage and guide adjuvant therapy decisions.^33^ Consistently,^34^ the current study indicated that patients with unstaged nodal status showed superior OS to those with LNM but inferior OS to those with negative nodal status (32 vs. 20 vs. 55 months, *P* = 0.001, Fig. 5), likely alluding to the underestimation of staging of a subset of patients without LND. Patients with unstaged nodal status are actually a combination of negative and understaged positive nodal patients. Indeed, the incidence of occult LNM in ICC patients without preoperative evidence of LNM was reported to be as high as 32.3–40.6%.^18, 29^ Of note, TNLE affects the odds of identifying LNM (Table 2). Consistent with AJCC recommendations,^35^ harvesting at least 6 LNs had the greatest discriminatory ability to identify LNM (Fig. 2). Accurate staging of nodal status contributes to guiding postoperative management, considering that patients with LNM are likely to benefit from adjuvant chemotherapy (Fig. 6A). The results were consistent with Qiao et al.’s study.^18^

The performance of LND for elderly ICC patients should involve considering not only the accuracy of staging information but also the risk of perioperative mortality and the benefit of long-term survival. In this nationwide cohort of elderly ICC patients aged ≥ 70 years, the total perioperative mortality was 2.6%, which is similar to that in Hiroko et al.’s study.^31^ Neither before nor after PSM was a significant difference in perioperative mortality observed between the LND group and the nLND group. Similarly, Zorays et al. reported that the perioperative mortality of ICC patients who underwent simultaneous LND or not was 5.7% and 5.9%, respectively.^28^ In particular, both the total adverse events and life-threatening complications related to LND were comparable among the LND and nLND groups.^18, 36^ As such, the risk of implementing LND for elderly ICC patients is manageable.

Regarding long-term survival, the median OS in the LND group was significantly longer than that in the nLND group (44 vs. 32 months, *P* = 0.045; Fig. 3B), which was contrary to the aforementioned meta-analysis.^16^ Notably, the studies included in the meta-analysis were all retrospective and mostly single-center, with great heterogeneity in population characteristics.^13^ Namely, comparisons between patients who did and did not receive LND were often unmatched. In general, patients who received LND were at high risk for LNM with a poorer prognosis.^28^ Unbalanced population characteristics could probably minimize the survival differences of the two populations.^18, 37^ In the current well-matched study, elderly patients who underwent LND showed superior OS to those who did not, and LND was demonstrated to be an independent protective factor for OS (Table 3). By further subgroup analysis, we found that elderly patients with relatively low tumor-burden characteristics and well-preserved liver function were more likely to benefit from LND (Fig. 4). In patients with the above features, the probability of systemic spreading^29^ and postoperative complications^24^ is lower. Thus, LND can be employed safely to remove lesions confined to the liver and regional lymph nodes. Notably, patients with extremely advanced age (> 80 years) might not benefit from LND. Older patients (> 80 years) are highly heterogeneous, are in poor general condition, have uncertain efficacy for surgery, and are rarely included in randomized clinical trials.^38^ In addition, the average life expectancy for Americans in 2020 will be approximately 77 years.^39^ For patients aged > 80 years, the potential benefit of LND for improving prognosis is probably discounted by the natural aging process itself.

To further investigate the significance of TNLE, unexpectedly, we found that AD-LND could not improve OS (mOS, 41 vs. 47 months, *P* = 0.612) but increased the odds of positive node retrieval by 6.49-fold (95% CI 1.98–21.28; *P* = 0.002) with respect to inAD-LND (Table 2). Thus, the prognostication of LND may be partly attributed to the definite evaluation of nodal status. Conversely, patients with AD-LND showed a longer median OS than inAD-LND patients in the LNM group (56 vs. 9 months, *P* = 0.043, supplementary 1). Similarly, Sahara et al. evaluated the therapeutic index of TNLE > 6 in node-positive patients with a value of 17.8 and indicated prolonged cancer-specific survival in node-positive patients with ≥ 3 LNs examined, which seems to confirm a survival benefit provided by LND.^19^ In contrast, several studies demonstrated the presence of LNM symbolizing a systemic disease in which the survival benefit of surgical treatment was limited.^40^ The exact prognostic value and appropriate candidates for LND need to be further confirmed in well-designed prospective multicenter studies.

This study has some limitations. First, owing to its retrospective nature, some biases could not be completely excluded between the two groups, even though a well-matched PSM was conducted. In this national cohort, perioperative management and surgical procedures, including the extent of LND and postoperative chemotherapy regimen, varied from each center, and detailed data on surgical margins, and tumor biomarker levels were not recorded. The results should be interpreted with caution given the missing clinical information. Even though adequate dissection was defined as harvesting at least 6 LNs in the current study, it may not exactly correspond to LND in the ideal sense. In the future, prospective well-designed multicenter trials should be conducted to further confirm the clinical benefit of LND we observed.

In conclusion, advanced age (≥ 70 years) was not a contraindication for LND, which facilitates accurate nodal staging and guides postoperative management. Appropriately selected elderly populations could benefit from LND without increasing perioperative mortality. Notably, the purpose of the procedure should be weighed against its limited prognostic benefit when LND is performed in extremely elderly patients (> 80 years).

### Supplementary Information

Below is the link to the electronic supplementary material.

Supplementary file1 (TIF 23415 KB)

## Data Availability

The SEER database is publicly accessible (https://seer.cancer.gov/).
