# Supplementary figures and images for: Is Lymphadenectomy Reasonable for Elderly Intrahepatic Cholangiocarcinoma Patients?
Source: J Gastrointest Surg. 2023 Oct 2;27(11):2451–63. doi: 10.1007/s11605-023-05846-y (PMC10661814; doi:10.1007/s11605-023-05846-y)

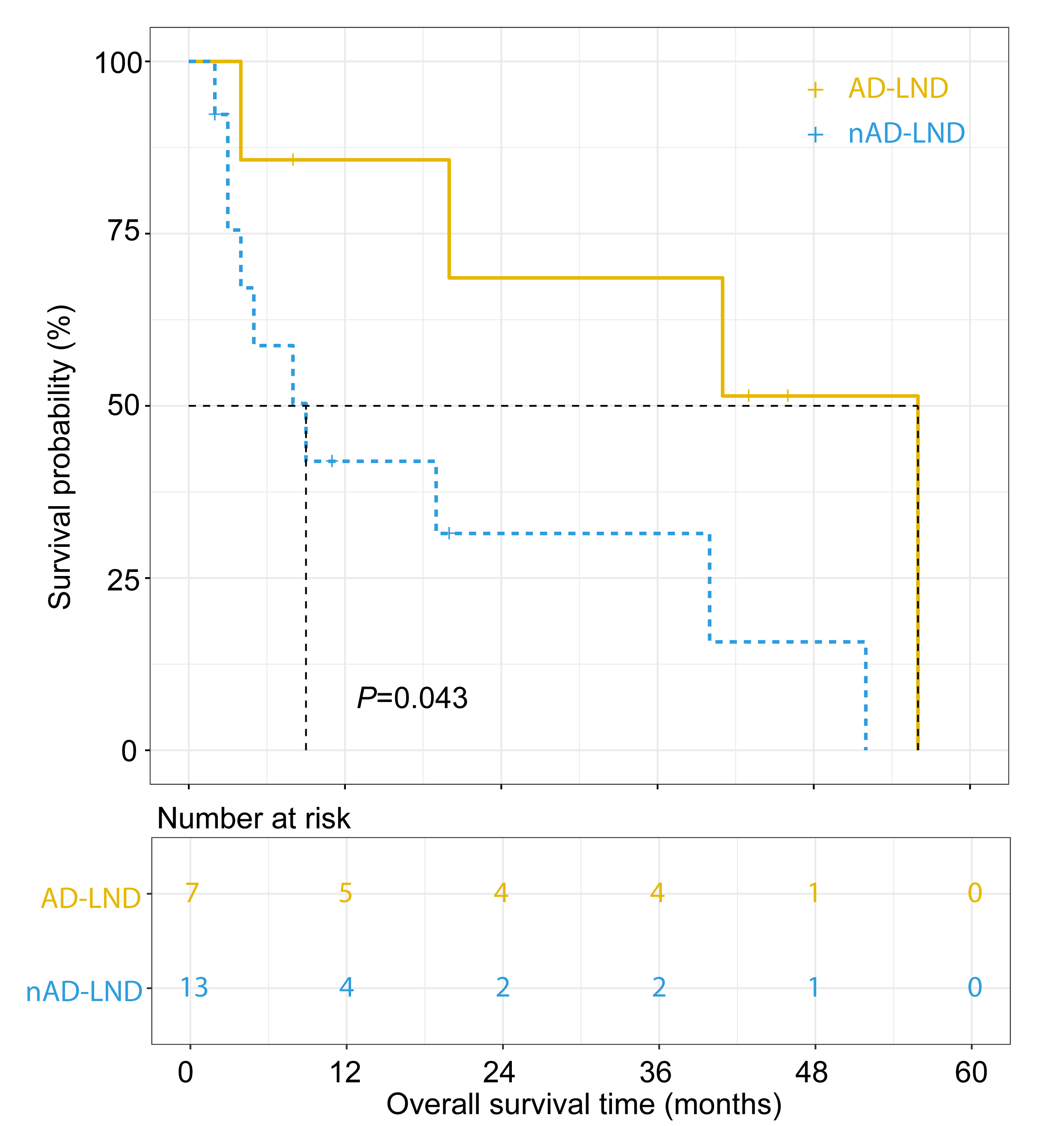

Supplement: Supplementary file 1 — Supplementary file1 (TIF 23415 KB) [file 11605_2023_5846_MOESM1_ESM.tif]
